# Minimally invasive approach to a deep-seated motor eloquent brain tumour: a technical note

**DOI:** 10.1093/jscr/rjab611

**Published:** 2022-01-21

**Authors:** Jose Pedro Lavrador, Anna Oviedova, Noemia Pereira, Sabina Patel, Kapil Mohan Rajwani, Priya Sekhon, Richard Gullan, Keyoumars Ashkan, Francesco Vergani, Ranjeev Bhangoo

## Abstract

Deep-seated brain tumours represent a unique neurosurgical challenge as they are often surrounded by eloquent structures. We describe a minimally invasive technique using tubular retractors and intraoperative neurophysiology monitoring for open biopsy of a deep-seated lesion surrounded by the corticospinal tract. We used preoperative functional mapping with diffusion tensor imaging tractography and navigated transcranial magnetic stimulation to identify a safe surgical corridor. We also used 5-Aminolevulinic Acid induced fluorescence to identify the lesion intraoperatively and optimize tissue samples obtained for histopathological diagnosis. We found the use of these tools improved the safety of surgery and reduced the risk of surgical morbidity.

## INTRODUCTION

Deep-seated brain tumours represent a unique neurosurgical challenge as they are often related to eloquent areas of the brain. Traditionally, access to deep-seated lesions would require brain retraction for adequate exposure, however this is associated with secondary brain damage. More recently, minimally invasive approaches to access deep-seated lesions have been described. The use of multimodal functional mapping, including navigated transcranial magnetic stimulation (nTMS), diffusion tensor imaging (DTI) tractography and intraoperative neurophysiology monitoring (IONM), has proved to be useful adjuncts helping surgeons preserve functional integrity and maximize resection. The integration of these multiple modalities for cortical and subcortical functional mapping has been shown to favour a better post-operative outcome [1–5]. We used these adjuncts to perform an open biopsy of a deep-seated lesion surrounded by the corticospinal tract (CST) using minimally invasive tubular retractors.

**Figure 1:**
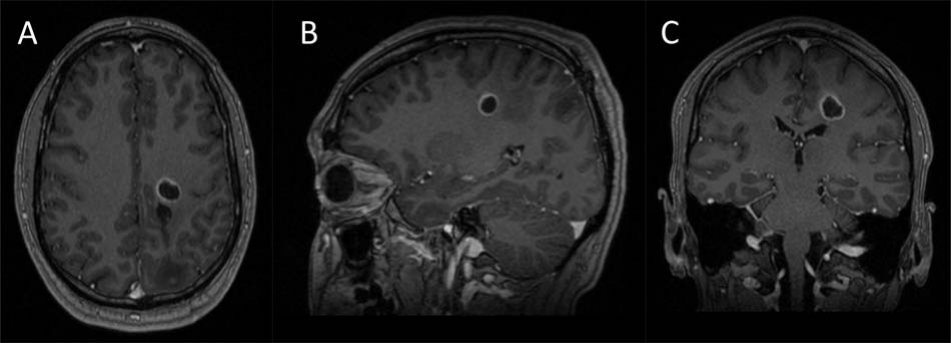
Preoperative imaging; (**A**–**C**) axial, sagittal and coronal T1-wieghted magnetic resonance imaging (MRI) images, with gadolinium demonstrating a contrast enhancing lesion in the left corona radiata.

**Figure 2:**
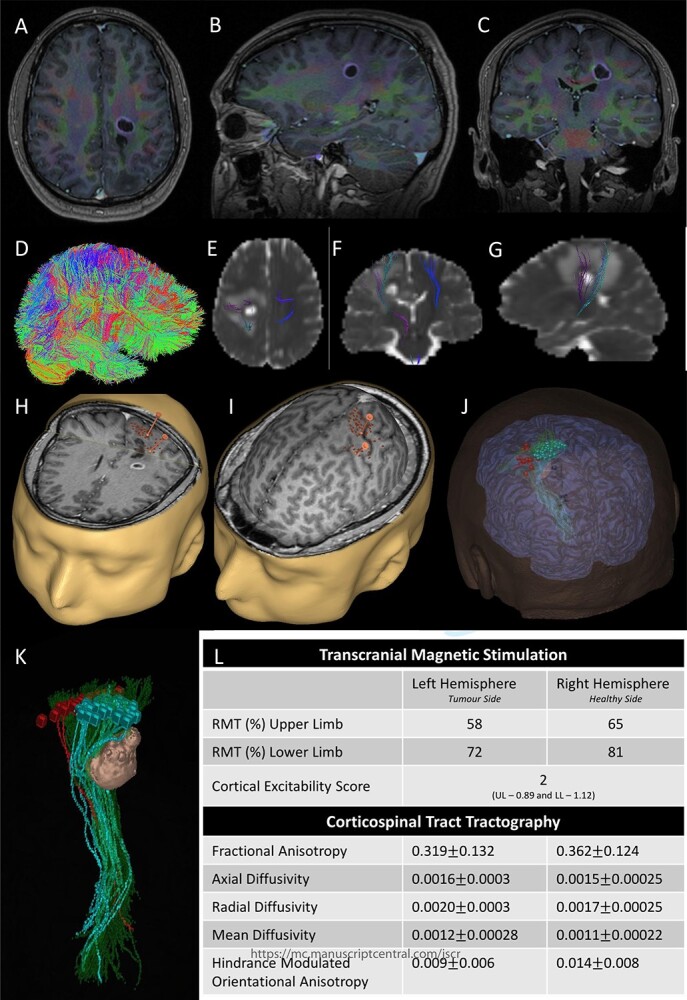
Integrated preoperative anatomical and functional brain mapping; (**A–C**) axial, sagittal and coronal fusion of T1-weighted MRI images with gadolinium and first eigenvector fractional anisotropy (FEFA); (**D**) whole brain tractography performed with StarTrack and visualized in TracViz according to deterministic spherical deconvolution algorithm. (**E–G**) fusion of CST with the ADC map; (**H** and **I**) nTMS mapping of the upper and lower limbs with hotspots for both upper and lower limbs identified with a marker (orange); (**J** and **K**) 3D Modelling of the tumour and the cortical and subcortical mapping of the CST with Stealth S8; (**L**) table summarizing the nTMS variables (resting motor threshold—RMT—and Cortical Excitability Score—Number of abnormal interhemispheric RMT ratios) and the tractography metrics.

## CASE REPORT

A 42-year-old, right-handed gentleman presented with 1-month history of right foot weakness and focal seizures. Imaging revealed a 1.6 × 1.4 × 1.6 cm enhancing lesion in the left corona radiate (Fig. 1). He underwent stereotactic frameless needle biopsy using the method previously described by our group [6]. The biopsy result was inconclusive, the procedure was uneventful and he recovered without neurological deficit. Post-operative computed tomography (CT) showed the biopsy site was posterolateral to the lesion and following discussion in our neuro-oncology multidisciplinary team meeting the consensus was to perform an open biopsy.

Preoperative cortical and subcortical motor mapping was performed (Fig. 2). Diffusion tensor and spherical deconvolution tractography was used to delineate the anatomy of the CST bilaterally. Tensor-derived metrics were calculated for both CSTs. The fractional anisotropy and the hindrance-modulated orientational anisotropy of the left CST (tumour side) were lower. The axial, radial and mean diffusivities were higher in the left CST. Navigated TMS (NEXSTIM©; single pulse technique) was used to assess CST function. Abnormal interhemispheric RMT ratios (iRMTr) were found for both upper and lower limbs and an abnormal combined cortical excitability score (number of abnormal iRMTr)—2/2. This information suggested altered cortical excitability and microstructure of the CST on the side with the tumour.

Two independent techniques were used to dissect the ipsilateral CST with StealthViz Software (MEDTRONIC©): region of interest (ROI) technique and TMS-seeded technique. In the first one, two ROIs were defined, precentral gyrus and midbrain at the level of the superior cerebellar peduncle, the anatomical streamlines going through both regions were selected. In the second technique, the positive nTMS responses for the upper and lower limb were selected independently as ROIs and the other ROI was in midbrain at the level of the superior cerebellar peduncle. Both dissections of the CST were used as they provided an anatomical and functional assessment of the tract. The tumour was delineated in the Cranial Software (MEDTRONIC©) and the preoperative cortical and subcortical mapping was integrated in a 3D model (Fig. 2).

**Figure 3:**
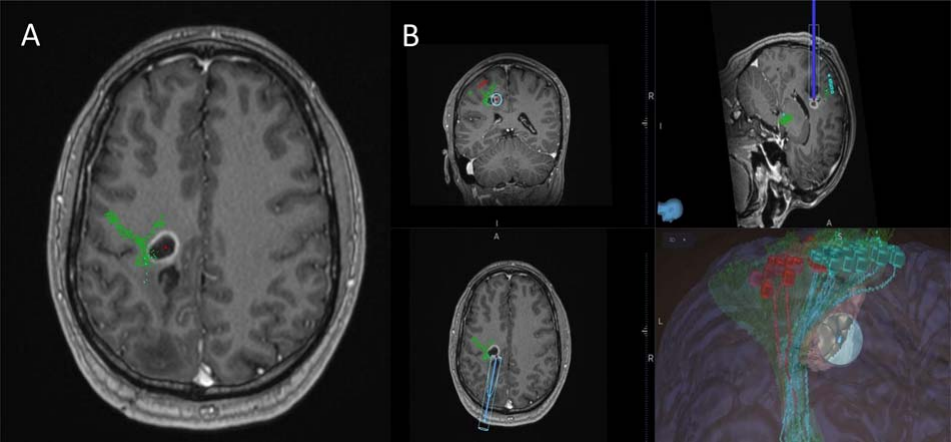
(**A**) Axial T1-weighted image with gadolinium showing the lesion with imposed DTI tractography of the CST; (**B**) planned trajectory for insertion of the tubular retractor guided by the preoperative integrated anatomical and functional motor mapping.

**Figure 4:**
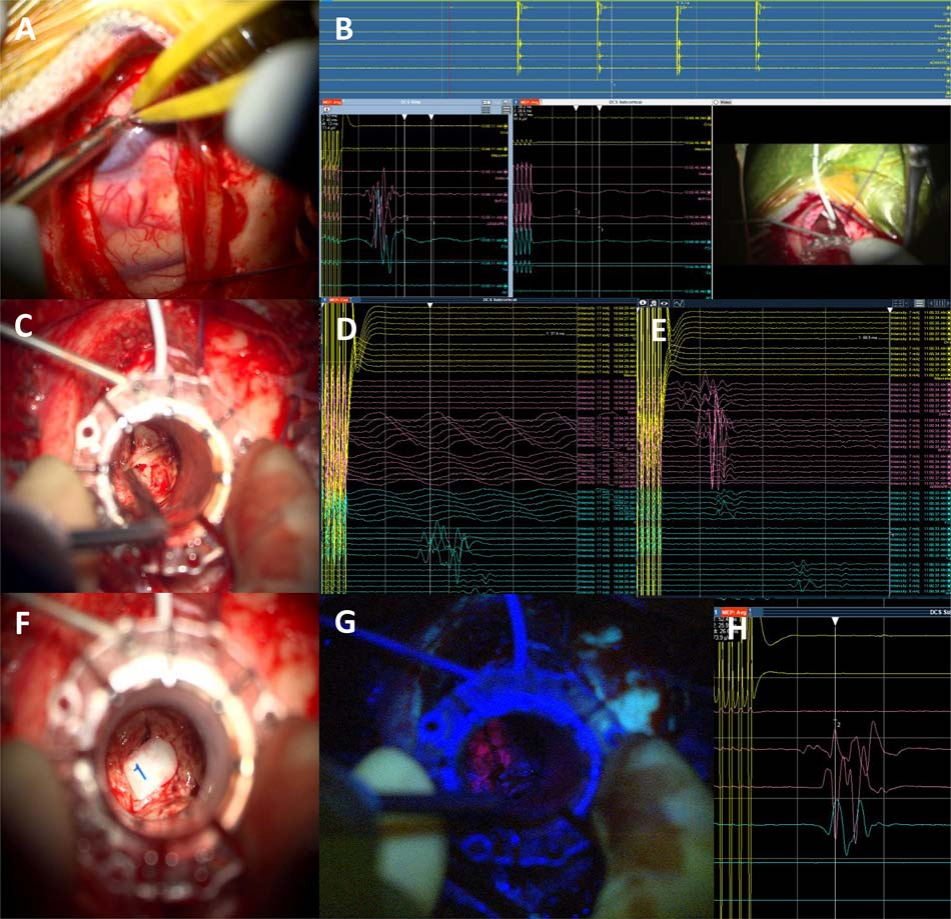
Intraoperative technical illustration: (**A**) transsulcal approach; (**B**) subdural strip of electrodes placed under preoperative nTMS and DTI guidance with stable responses at 7 mA; (**C**) docking of the BrainPath tubular retractor with positive responses of the *anterior tibialis* at 17 mA during the cannulation of the brain, insertion stopped at this point; (**D**) stimulation through the tube identified motor responses at 17 mA from lower limb muscles (tibialis anterior and abductor hallucis); (**E**) direct subcortical stimulation identified motor responses from upper and lower limb muscles at 7 mA; (**F** and **G**) Tumour subtotal resection stopped at 5 mA for the upper limb and 7 mA for the whole CST. The tumour demonstrated 5-ALA-induced fluorescence under the BLUE 400 filter; (**H**) activity from hand muscles at 5 mA threshold during removal of BrainPath tubular retractor before closure.

5-Aminolevulinic Acid (5-ALA) was given orally 2 h before the surgery. The least disruptive trajectory was selected taking into account the previous biopsy and the preoperative mapping information (Fig. 3). Under Stealth guidance, a skin incision was made overlying the planned craniotomy site. After the craniotomy, the dura was opened and a subdural strip of electrodes was placed over the primary motor cortex. Replicable motor responses from the hand muscles were obtained at 7 mA current intensity (Fig. 4). Motor evoked potentials (MEPs) were obtained continuously throughout the procedure. A transsulcal parafascicular approach was used. The sulcus was opened sharply under the microscope. At the depth of the sulcus, a preselected tubular retractor (NICO BrainPath 75 mm × 13 mm) was passed to the superficial surface of the lesion*.* While performing brain cannulation to the lesion, a monopolar probe (INOMED©) navigated with SureTrack (MEDTRONIC©) was used to perform continuous subcortical stimulation, train-of-five technique using high-frequency stimulation.

Once the superficial aspect of the lesion was reached, positive stimulation of the lower limb was identified at 17 mA (Fig. 4). The tubular retractor was fixed with a Leyla retractor system and held manually to prevent displacement. Multiple tissue samples were obtained from 5-ALA-induced fluorescent tissue. Intraoperative smear was consistent with high-grade lesion. After the biopsy was performed the tubular retractor was slowly removed. Using a monopolar probe for subcortical stimulation directly through the surgical site, we were able to confirm the proximity to the CST at 5 mA to the lower limb motor fibres. Cortical MEPs were stable during the whole procedure.

**Figure 5:**
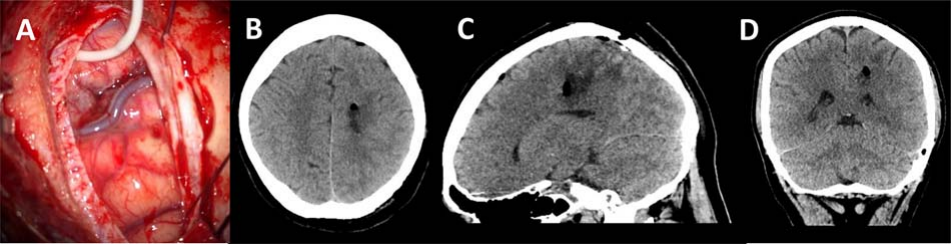
Post-operative imaging; (**A**) post-decannulation brain surface after transsulcal approach; (**B–D**) axial, sagittal and coronal CT images showing collapse of the surgical tract and no overt complications.

A post-operative CT head was performed within 24 h of surgery documenting collapse of the surgical tract (Fig. 5). The patient did not have any post-operative deficit and was discharged home. Histopathology confirmed a Glioblastoma Multiforme.

## DISCUSSION

The conventional approach to deep-seated tumours has been to perform a stereotactic needle biopsy to obtain tissue for diagnosis, followed by adjuvant therapy [7]. However, stereotactic biopsies can be non-diagnostic in 2–15% of cases [8]. Cystic lesions, in particular, represent a diagnostic challenge as the needle may not be able to penetrate the wall of the lesion. As seen in this case, the use tubular retractor systems are a viable alternative. Tubular retractors allow the gentle splitting of brain fibres and are associated with reduced risk of secondary brain injury [9].

Preoperative and intraoperative functional mapping has been widely described in resection surgery of infiltrative gliomas to preserve functional integrity and this has shown to improve long-term outcomes [10–14]. In this case, the preoperative nTMS documented a functional impairment of the primary motor cortex and CST and tractography revealed altered white matter microstructure of the CST [15]. This information was important as it indicated an injured motor pathway more susceptible to non-reversible injury [16]. Identifying the anatomical location of the of the CST allowed for a tailored surgical approach, avoiding cortical and subcortical injury to the motor pathway. We used IONM, cortical and subcortical stimulation, during brain cannulation and at depth of the tubular retractors while taking biopsies to keep a safe margin away from the CST.

We found preoperative and intraoperative functional motor mapping, using DTI, nTMS and IONM, and 5-ALA to be useful adjuncts when performing this open biopsy using tubular retractors of a deep-seated lesion in a highly eloquent area of the brain.

## CONFLICT OF INTEREST STATEMENT

None declared.

## FUNDING

None.
